# Novel variant of the *NCSTN* gene identified in a woman with hidradenitis suppurativa

**DOI:** 10.1111/ddg.15792

**Published:** 2025-06-04

**Authors:** Conrad Hempel, Sonja Grunewald, Till Mittank‐Weidner, Jan‐Christoph Simon, Franziska Schnabel, Robin‐Tobias Jauss, Viktor Schnabel

**Affiliations:** ^1^ Department of Dermatology Venereology und Allergology University Medical Center Leipzig Leipzig Germany; ^2^ Institute of Human Genetics University Medical Center Leipzig Leipzig Germany

**Keywords:** familiar acne inversa, Hidradenitis suppurativa, *NCSTN* gene mutation

Dear Editors,

A Caucasian 27‐year‐old female with a 4‐year history of hidradenitis suppurativa presented to our outpatient clinic. She reported undergoing repeated extensive surgical procedures for the removal of dermal abscesses in the axillae and groin, which, interestingly, also appeared in uncommon locations such as the trunk and thighs. Prior to this, she had been treated intermittently with antibiotics, isotretinoin, and a contraceptive pill. Additionally, anti‐TNF‐α antibody therapy had been administered for 6 months, and most recently, treatment with an anti–IL‐17 agent had been given for 3 months. All medications had been discontinued, either due to side effects or lack of efficacy.

She mainly complained of severe pain caused by recurrent abscesses, which resulted in an inability to work. She denied nicotine use and had a BMI of 18.5. Due to comorbid depression and anxiety disorder, she was receiving psychological support. Her *Dermatology Life Quality Index* (DLQI) score was 29.

Dermatological examination showed many inflammatory papules, comedones and pustules distributed over her whole body with predominant areas of face trunk und upper legs being involved. Abscess formations up to 2 cm diameter could be found in both axillae, in the pubic and gluteal area (Figure 1a,b), corresponding to Hurley grade 2. Interestingly, both her mother and maternal uncle had a history of recurrent painful abscesses, though to a lesser extent (online supplementary Figure S1).

**FIGURE 1:**
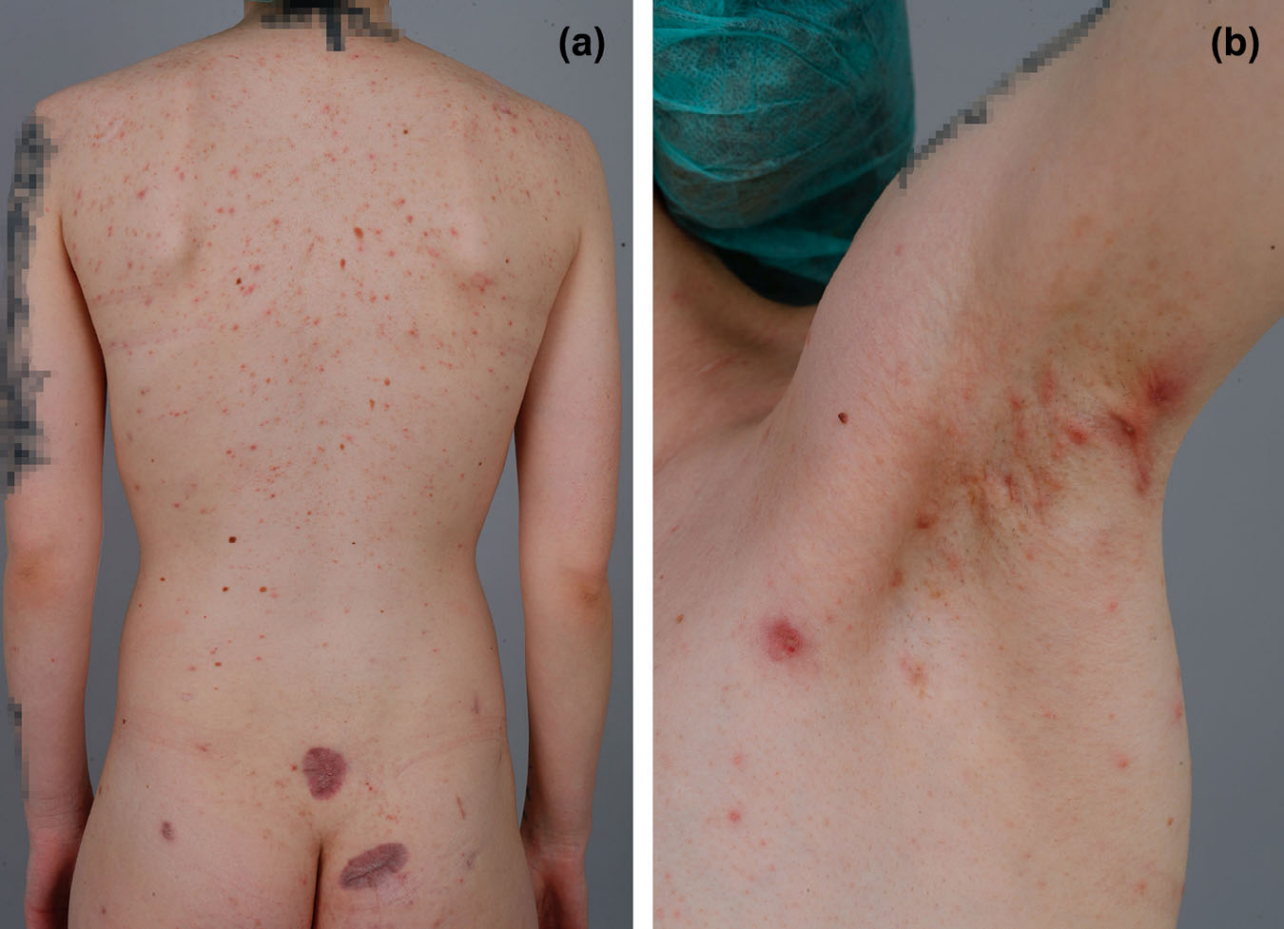
a) Back and b) left axilla of a 27‐year‐old woman with familial HS caused by *NCSTN* variant showing multiple inflamed papules and comedones disseminated all over the trunk. Two flat scars on the lower back/buttocks from previous abscess excisions.

Because of the positive family history, we performed whole exome sequencing using TWIST Human Core Exome Kit (TWIST Bioscience, San Francisco, USA) on an Illumina NovaSeq6000 sequencer (Illumina, San Diego, USA). Analysis using the browser‐based genomics software Varvis (Limbus Medical Technologies GmbH, Rostock, Germany) identified a heterozygous truncating variant in the *NCSTN* gene (NM_015331.3:c.1101_1101+17delinsTGTCCA, p.(Gln367Hisfs*6)) (online supplementary Figure S2). We obtained a mean coverage of the *NCSTN* gene of 79.49 times and the variant was confirmed in more than 50 reads with a quality score of 1918 (GATK HaplotypeCaller) and no conflicting reads in the area. For confirmation, Sanger sequencing was performed (online supplementary Figure S3). The variant causes a frameshift and premature protein truncation, but has not been reported in variant databases (HGMD, Decipher, ClinVar) or described in the literature. The variant is also absent from the general population database gnomAD, which includes sequencing data from over 1,150,000 European (non‐Finnish) controls at the variant position. According to ACMG Classification the variant was classified as pathogenic (criteria applied: PVS1, PM2_SUP, PP4).^1^ Unfortunately, other family members were not available for segregation analysis.

Hidradenitis suppurativa (HS), also known as Acne inversa, is a chronic, inflammatory, recurrent disease of the hair follicle that usually presents with painful, inflamed lesions, most commonly in the axillar, inguinal and anogenital region.^2^ The prevalence in Europe is about 1%.^2^ The typical age range for the onset of HS is generally between 20 and 40 years.^3^ Approximately 40% of cases can be classified as familial, in addition to syndromic and sporadic variants.^3^ Familial HS is generally characterized by more severe symptoms and earlier age of onset.^3^ In the literature, at least four different genes of the γ‐secretase complex have been reported as causative for HS, including *NCSTN*, *PSENEN*, *PSEN1*, and *APH1B*.^4^, ^5^ *NCSTN* encodes Nicastrin, a type‐1 transmembrane glycoprotein of the y‐secretase complex, which plays a central role in Notch signaling pathway and controls anti‐proliferative and differentiation‐promoting effects in human keratinocytes. Knockdown of *NCSTN* leads to increased proliferation and decreased differentiation of keratinocytes, primarily mediated through the Notch and PIK‐AKT signalling pathways.^6^ So far, 52 variants in *NCSTN* have been described in HS.^7^ A monogenic variation in *NCSTN* is linked to an earlier onset and atypical presentation at uncommon skin sites such as the torso. Furthermore, low body weight and an increased likelihood of treatment with biological therapy distinguish this genetic variant from classic HS, as confirmed by this case.^7^ The interaction between genetic factors and dysregulation of immune mediators such as TNF‐α, IL‐1β, IL‐17, and IL‐12/23 contributes to the chronic inflammatory nature of HS.^3^ Potential therapeutic targets are therefore anti‐TNF‐α, anti‐IL‐1α, anti‐IL‐17, and anti‐IL‐12/23 therapies as well as JAK inhibitors.

This case provides further evidence regarding the functional implication of *NCSTN* variants in the pathogenesis of familial HS, which has been described mainly in Asian populations.^8^, ^9^

In our patient we described a novel truncating *NCSTN* variant leading to HS. Interestingly, no environmental exacerbating factors, such as obesity or smoking could be identified in our patient. Clinically, the phenotype belongs to the rarer “follicular” subtype of HS, which was described by Canoui‐Poitrine et al as one of three subtypes.^10^ It is mainly characterized by follicular lesions, such as comedones, epidermal cysts, sinus pilonidalis and acne vulgaris and shows a more severe course of disease than the regular axillary/groin type. It should be noted that the comedones observed in our patient cannot solely account for the severe destructive inflammation. Considering the genetic background, this report provides further evidence that HS can no longer be considered a simple “disease of follicular occlusion”. The significant role of inflammation and innate/adaptive immunity in familiar HS cannot be overstated and raises the question whether follicular occlusion is a primary or secondary phenomenon in the pathogenesis of the disease.

Our case report broadens the genetic spectrum associated with HS. In combination with the clinical phenotype, it provides valuable information for future studies on treatment strategies and surveillance programs in affected families.

## CONFLICT OF INTEREST STATEMENT

None.

## Supporting information



Supplementary information

Supplementary information

Supplementary information
