# Supplementary figures and images for: Novel variant of the NCSTN gene identified in a woman with hidradenitis suppurativa
Source: J Dtsch Dermatol Ges. 2025 Jun 4;23(10):1332–4. doi: 10.1111/ddg.15792 (PMC12548324; doi:10.1111/ddg.15792)

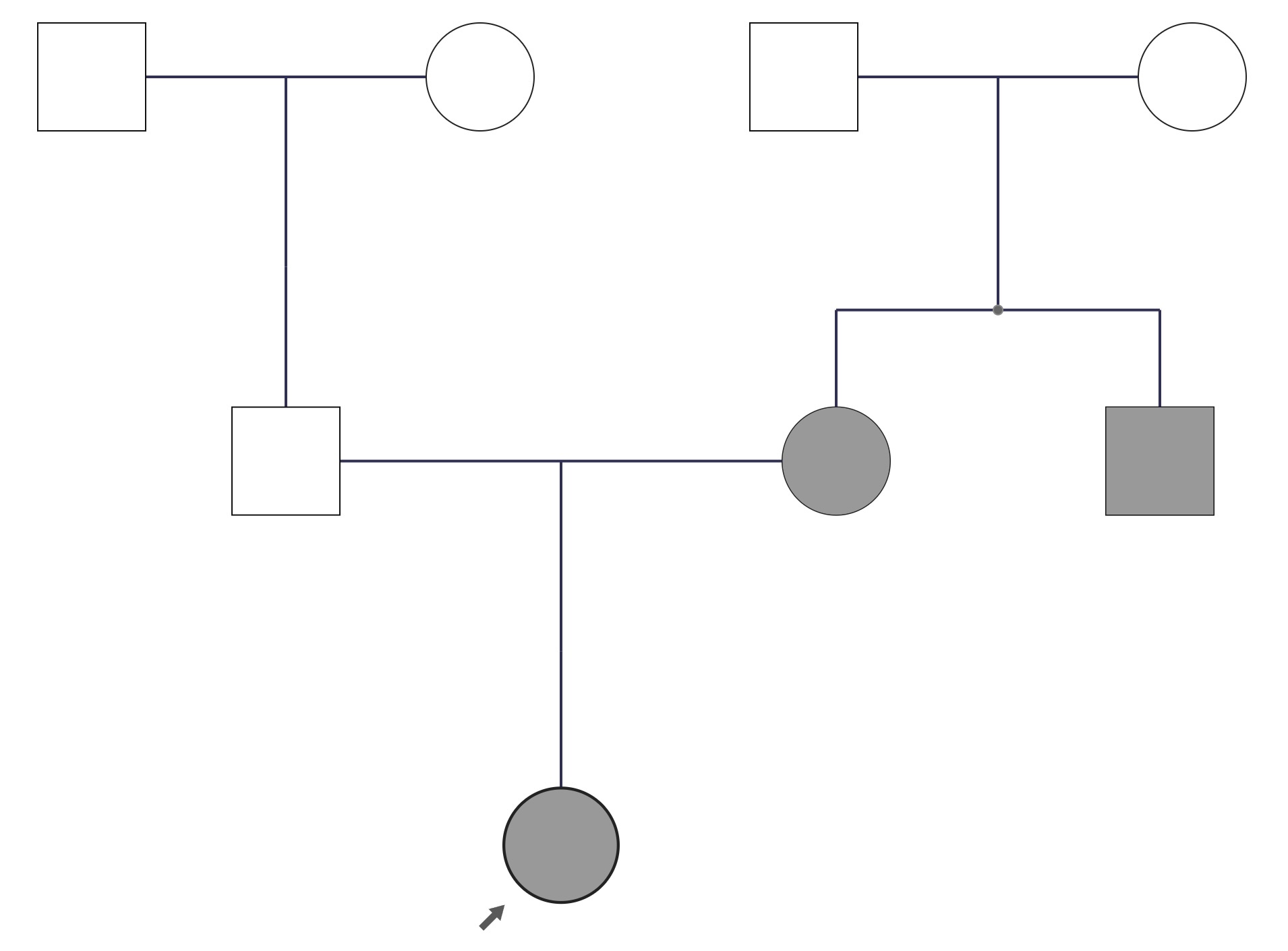

Supplement: Supplementary file 1 — Supplementary information [file DDG-23-1332-s002.jpg]

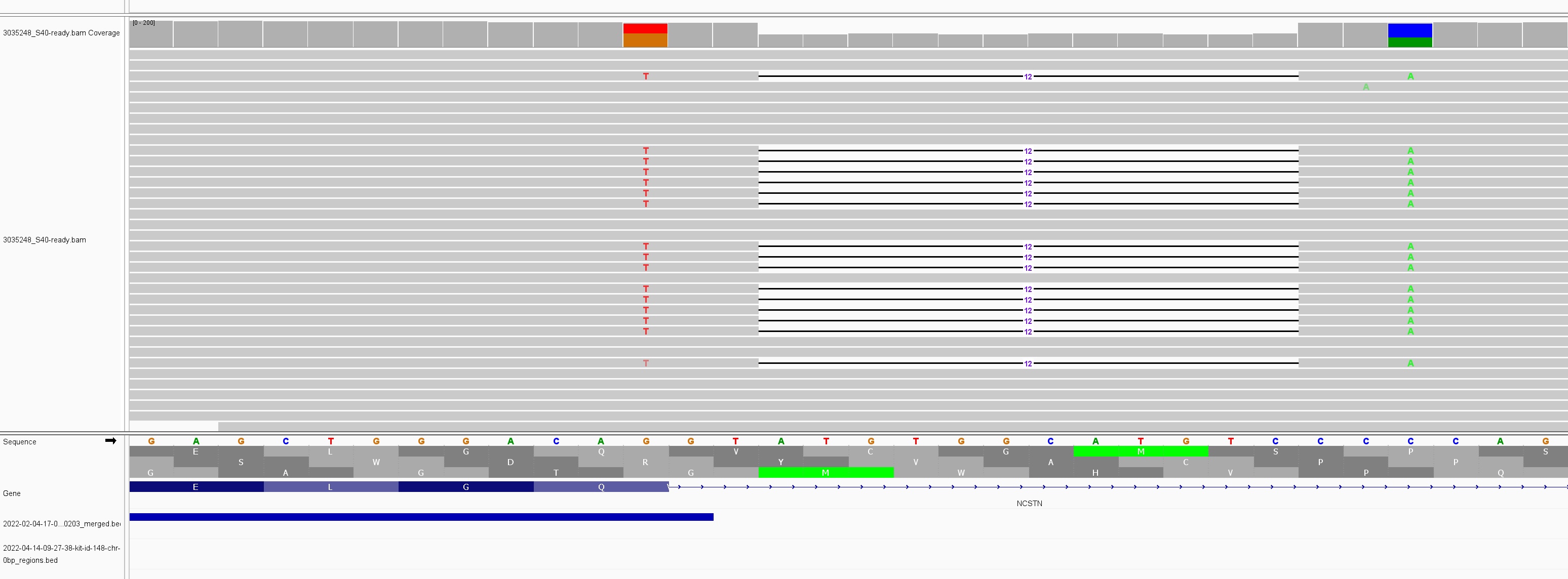

Supplement: Supplementary file 2 — Supplementary information [file DDG-23-1332-s003.jpg]

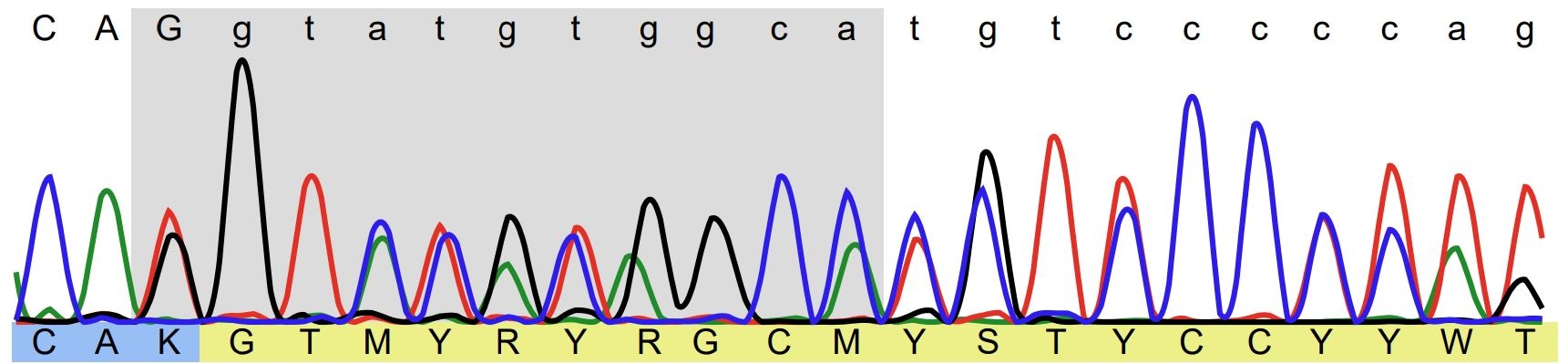

Supplement: Supplementary file 3 — Supplementary information [file DDG-23-1332-s001.jpg]
